# Second Scope, New Findings: Pediatric Stridor Is Not Always Due to Croup or Laryngomalacia: A Case Report

**DOI:** 10.5811/cpcem.38443

**Published:** 2025-06-19

**Authors:** Summer Ghaith, Deborah Hsu, William Dixon

**Affiliations:** * Mayo Clinic Alix School of Medicine, Mayo Clinic, Phoenix, Arizona; † Stanford University School of Medicine, Department of Emergency Medicine, Division of Pediatric Emergency Medicine, Palo Alto, California; ‡ Stanford University School of Medicine, Department of Emergency Medicine, Palo Alto, California

**Keywords:** infantile subglottic hemangioma, subglottic stenosis, stridor, respiratory distress, case report

## Abstract

**Introduction:**

Infantile subglottic hemangioma is a rare and serious condition characterized by stridor, respiratory distress, and a barking cough. This condition poses a significant risk as it can lead to life-threatening airway obstruction.

**Case Report:**

We present a five-week-old patient who was diagnosed in the emergency department (ED) with moderate laryngomalacia via laryngoscopy by otolaryngology and discharged; he returned to the ED the next day with worsening symptoms of recurrent stridor, difficulty feeding, and worsening respiratory distress. A second laryngoscopic exam performed on the return ED visit revealed a subglottic mass that was later identified as a left-sided subglottic hemangioma via bronchoscopy and magnetic resonance imaging. The patient was treated with propranolol and discharged from the inpatient unit with dermatology and otolaryngology follow-up.

**Conclusion:**

Infantile subglottic hemangioma is a rare but serious cause of respiratory distress in infants, posing a risk of airway obstruction. This diagnosis should be considered in the ED, particularly for patients under two years of age, who present with recurrent stridor and respiratory distress and do not respond to standard treatments for croup.

## INTRODUCTION

Infantile subglottic hemangioma is a rare and serious condition that is potentially life-threatening.^1^ It is a benign vascular tumor of infancy that, if large enough, could obstruct the airway. A mortality rate of up to 50% has been reported in this circumstance.^2^,^3^ Infantile hemangiomas have an incidence of 4%–5%, but involvement of the subglottic region is much more rare.^1^ Subglottic hemangioma accounts for approximately 1.5% of congenital laryngeal abnormalities.^1^ Most patients will present with sudden-onset symptoms including dyspnea, stridor, barking cough, hoarseness, respiratory distress, feeding difficulty, and cyanosis.^1^,^4^ Given the nature and presentation of these symptoms, it is commonly confused with croup. We present a case of a patient diagnosed with a left-sided subglottic hemangioma on a subsequent visit to the emergency department (ED) after an initial diagnosis of moderate laryngomalacia.

## CASE REPORT

A five-week-old male patient born at 35 weeks gestational age, presented to the pediatric ED for evaluation of difficulty breathing and stridor. Parents noted he had never experienced stridulous breathing previously. He had been well the night before the onset of acute symptoms, and his birth history was unremarkable. On the first visit, his vital signs were heart rate 190 beats per minute (bpm), respiratory rate 56 breaths per minute, temperature 37.4 ºCelsius, and oxygen saturation 99% on room air. Review of symptoms was unremarkable. An extended viral panel was performed, which included influenza A, influenza B, respiratory syncytial virus, coronavirus disease 2019, parainfluenza, metapneumovirus, rhinovirus, enterovirus, chlamydia pneumoniae, and mycoplasma pneumoniae. These were all negative. He had some but not complete symptomatic improvement with racemic epinephrine and oral dexamethasone.

Otolaryngology was consulted, and a laryngoscopic exam was performed in the ED. The exam demonstrated moderate laryngomalacia, but prior to this diagnosis he required two additional doses of racemic epinephrine during his ED stay. He was admitted to the acute care pediatric unit overnight for observation. He required no further interventions during the hospital stay and was discharged within 12 hours of admission. On discharge, his vital signs were heart rate 156 bpm, respiratory rate 21 breaths per minute, temperature 36.8 ºCelsius, and oxygen saturation 99% on room air. Twelve hours after discharge from the hospital, the patient developed worsening inspiratory and expiratory stridor with feeds and crying, increased fussiness, and could not finish his feeding.

The patient’s presenting vital signs on his second ED visit included temperature 36.9 ºC, blood pressure 105/71 millimeters of mercury, heart rate 196 bpm, respiratory rate 26 breaths per minute, and oxygen saturation 100% on room air. The initial physical examination was notable for a crying, mottled infant in respiratory distress. Nasal congestion was present. There was increased work of breathing, as well as non-positional inspiratory stridor that worsened with crying or agitation. Grunting and suprasternal retractions were present. Biphasic stridor was not seen in the ED, but parents reported biphasic stridor at home. The rest of his physical exam was normal, including a skin examination that was negative for other cutaneous hemangiomas.

High-flow nasal cannula oxygen was started. Racemic epinephrine and dexamethasone were immediately started with some but not complete improvement of symptoms. The patient had decreased respiratory distress and improved color and overall condition after ED interventions. Labs were obtained. A complete blood count and comprehensive metabolic panel were unremarkable. Lactate was elevated to 2.73 millimoles per liter (mmol/L) (referencee range: 0.5 to 2.2 mmol/L). A chest radiograph (CXR) was performed, which showed diffuse bronchial cuffing. This was the same finding as the CXR from the first ED visit the day prior.

**Image 1:**
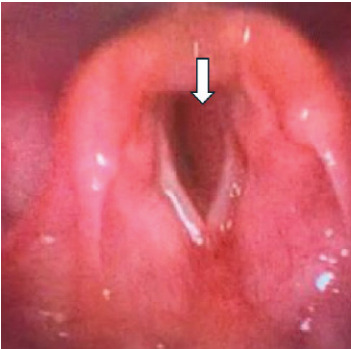
Laryngoscope finding of mild post-cricoid edema consistent with subglottic hemangioma (arrow).

A repeat laryngoscopic exam was performed in the ED by an otolaryngologist to explore other etiologies of the patient’s symptoms. Moderate laryngomalacia was again seen, but this time a mild post-cricoid edema (concerning for subglottic stenosis) was also found (Image 1).

**Image 2:**
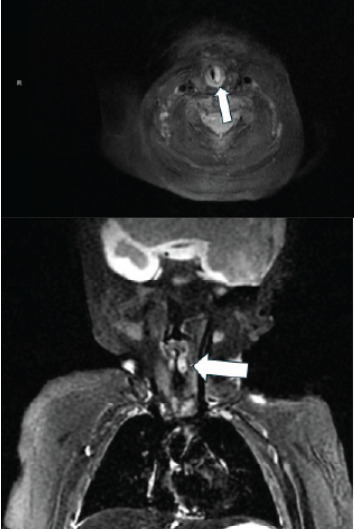
Neck and chest magnetic resonance imaging showing an enhancing lesion in the left subglottic larynx consistent with subglottic hemangioma, axial view above and coronal view below (arrows).

Neck and chest magnetic resonance imaging (MRI) was performed during the patient’s hospital admission. A well-circumscribed sub-centimeter enhancing lesion in the left subglottic larynx suggestive of a subglottic hemangioma was seen causing moderate narrowing of the subglottic airway (Image 2).

*CPC-EM Capsule*

What do we already know about this clinical entity?

*Infantile subglottic hemangioma is a rare, life-threatening airway lesion causing sudden stridor, dyspnea, barking cough, hoarseness, distress, and cyanosis*.

What makes this presentation of disease reportable?

*A patient was diagnosed with a left-sided subglottic hemangioma on a return emergency department visit after initially being diagnosed with moderate laryngomalacia*.

What is the major learning point?

*Consider infantile subglottic hemangioma in children under two with persistent stridor or poor response to airway treatments; propranolol is first-line therapy*.

How might this improve emergency medicine practice?

*Point-of-care laryngoscopy may expedite diagnosis and guide management in pediatric patients with upper airway obstruction, improving emergency care outcomes*.

The patient was admitted from the ED to the pediatric intensive care unit (PICU) for close monitoring of his airway with a plan to go to the operating room (OR) the following morning for bronchoscopy to assess the subglottic hemangioma as well as a possible supraglottoplasty for the patient’s diagnosed laryngomalacia. Upon admission to the PICU, his vital signs were heart rate 152 bpm, respiratory rate 39 breaths per minute, and oxygen saturation 100% on 3L/21% high-flow nasal cannula. After administration of racemic epinephrine and dexamethasone, high-flow nasal cannula oxygen was started in the ED for respiratory support and continued in the PICU. Heliox was also used in the PICU early in his hospitalization.

**Image 3:**
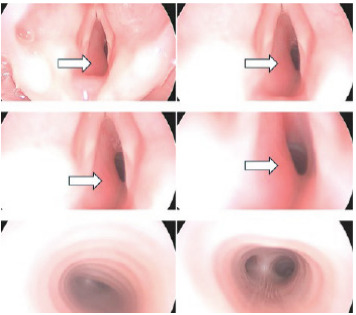
Operating room bronchoscopy illustrating subglottic hemangioma (arrows).

In the OR, the patient underwent bronchoscopy, which showed a narrowed subglottis due to a reddish mass emanating from the left subglottis, consistent with subglottic hemangioma (Image 3).

Dermatology was consulted for the patient’s new diagnosis of left-sided subglottic hemangioma. Infectious etiology of symptoms was deemed less likely than this diagnosis, and the patient was started on propranolol. He was scheduled for follow-up with pediatric otolaryngology and dermatology clinics for outpatient monitoring of his subglottic hemangioma.

## DISCUSSION

We present a case of a patient diagnosed with a left-sided subglottic hemangioma on a subsequent visit to the ED after an initial diagnosis of moderate laryngomalacia. Risk factors for infantile subglottic hemangiomas include factors such as female sex, prematurity, low birth weight, and family history of hemangioma.^5^ Drug exposures can increase the risk as well, including maternal beta-blockers, progesterone, and illicit drugs.^5^

Differential diagnoses for pediatric stridor can be organized by the patient’s age. In patients under six months of age, emergency physicians should consider laryngotracheomalacia, vocal cord paralysis, subglottic stenosis, airway hemangiomas, and vascular rings. In patients greater than six months of age, physicians should consider croup, epiglottitis, bacterial tracheitis, foreign body aspiration, and retropharyngeal abscess. In our case, our patient was initially diagnosed with laryngomalacia. Laryngomalacia is a common cause of stridor that presents with inspiratory stridor that worsens when feeding or when the patient is supine. This is a self-limiting disease and usually resolves within 12–24 months of age.^6^ Moreover, subglottic hemangioma is commonly misdiagnosed as croup. Airway problems should be considered in patients who are being evaluated for croup in cases of recurrent (two or more episodes per year) or prolonged symptoms.^7^ Additionally, close attention should be paid to infants younger than 12 months, infants with a history of intubation, and premature infants.^7^

Typically, infantile subglottic hemangiomas are not present at birth and develop within the first few months of life, followed by a proliferation phase of about 6–9 months, and subsequently a spontaneous regression phase that occurs over years.^2^ During the proliferative phase, the risk for airway obstruction is at its highest.^2^ Patients typically present during this proliferative phase with symptoms of stridor, feeding difficulties, and respiratory distress, as in our case. Stridor in these patients is characterized as a biphasic stridor associated with a barky cough that develops as the hemangioma enlarges. Additionally, subglottic hemangiomas are commonly associated with cutaneous findings of cutaneous hemangioma and segmental hemangiomas in a “beard distribution.”^8^ Diagnosis of infantile subglottic hemangioma is usually established with endoscopy.^3^ Imaging such as computed tomography (CT) and MRI can be used to determine the depth of the lesions or to exclude other etiologies.^3^

First-line treatment of subglottic hemangioma is propanolol.^3^ Propranolol is the drug of choice for subglottic hemangiomas as it has been shown to significantly reduce the size of hemangiomas and alleviate the symptoms of stridor and respiratory distress. Propranolol is a non-selective beta-blocker that causes capillary vasoconstriction, decreased expression of vascular endothelial growth factors to inhibit angiogenesis, apoptosis of capillary endothelial cells, and inhibition of nitric oxide production, causing the hemangioma to shrink.^8^ Dosage of propranolol is 2–3 milligrams per kilogram per day.^8^ One study found that stridor was eliminated with use of propranolol within 24 hours or less in 85% of patients.^9^ The recommended duration of treatment with propranolol is at least six months; however, continuing treatment until at least 12 months of age may reduce the risk of rebound growth.^8^ As dermatologists are typically the primary specialists involved in the treatment of cutaneous hemangiomas with propranolol, they are well-positioned to manage the cases associated with airway hemangiomas comprehensively. However, a multidisciplinary approach with pediatricians and otolaryngologists is warranted.

Other treatment modalities include steroids, both systemic and intralesional, alpha-interferon, vincristine, bleomycin, laser, tracheostomy, and surgical excision.^1^,^10^–^12^ However, propranolol is associated with a high rate of hemangioma clearance with an expected clearance of 95%, which is superior to other treatments and usually avoids the need for surgery.^8^

Infantile subglottic hemangioma is a critical pediatric emergency. An emergency physician should consider intubating an infant with subglottic hemangioma if the infant presents with severe respiratory distress, signs of impending respiratory failure, or if there is a rapid deterioration in the clinical condition.^8^ It is important to use a smaller endotracheal tube in a stridulous infant because the subglottic space is already narrowed due to the hemangioma.^13^ A smaller tube can help pass through the stenotic area while reducing the risk of trauma to the airway.^13^ As emergency physicians increasingly expand their scope of practice, developing competency in diagnostic laryngoscopy may be useful. The ability to promptly diagnose conditions such as infantile subglottic hemangioma has the potential to impact patient outcomes through timely and appropriate interventions.

## CONCLUSION

Infantile subglottic hemangioma is a rare cause of pediatric respiratory distress, but it is an important condition to consider when formulating differential diagnoses for pediatric patients with signs of upper airway obstruction. Specific patient populations in which to consider this diagnosis are children less two years of age who have recurrent or worsening stridor and other respiratory symptoms, and/or those who do not respond to standard treatment for upper and lower airway diseases. Propranolol remains first-line treatment for infantile subglottic hemangioma. This case illustrates the role point-of-care diagnostic laryngoscopy may have in shortening the time to diagnosis and informing management decisions of patients with signs of upper airway obstruction.
